# NF-κB-activated SPRY4-IT1 promotes cancer cell metastasis by downregulating *TCEB1* mRNA via Staufen1-mediated mRNA decay

**DOI:** 10.1038/s41388-021-01900-8

**Published:** 2021-06-23

**Authors:** Lin Zhao, Longyang Jiang, Ming Zhang, Qiang Zhang, Qiutong Guan, Yalun Li, Miao He, Jingdong Zhang, Minjie Wei

**Affiliations:** 1 Department of Pharmacology, School of Pharmacy, China Medical University, No.77 Puhe Road, Shenyang North New Area, Shenyang City, 110122 Liaoning China; 2 Liaoning Key Laboratory of molecular targeted anti-tumor drug development and evaluation, China Medical University No.77 Puhe Road, Shenyang North New Area, Shenyang City, 110122 Liaoning China; 3 Department of Anorectal Surgery, The First Hospital of China Medical University, Shenyang, China; 4 Medical Oncology Department of Gastrointestinal Cancer, Liaoning Cancer Hospital & Institute, Cancer Hospital of China Medical University, Shenyang, China

**Keywords:** Cancer, Cell biology

## Abstract

Previous study demonstrated that most long non-coding RNAs (lncRNAs) function as competing endogenous RNAs or molecular sponges to negatively modulate miRNA and regulate tumor development. However, the molecular mechanisms of lncRNAs in cancer are not fully understood. Our study describes the role of the lncRNA SPRY4 intronic transcript 1 (SPRY4-IT1) in cancer metastasis by mechanisms related to Staufen1 (STAU1)-mediated mRNA decay (SMD). Briefly, we found that, high SPRY4-IT1 expression was associated with aggressiveness and poor outcome in human colorectal, breast and ovarian cancer tissues. In addition, functional assays revealed that SPRY4-IT1 significantly promoted colorectal, breast and ovarian cancer metastasis in vitro and in vivo. Mechanistically, microarray analyses identified several differentially-expressed genes upon SPRY4-IT1 overexpression in HCT 116 colorectal cancer cells. Among them, the 3′-UTR of transcription elongation factor B subunit 1 (*TCEB1*) mRNA can base-pair with the Alu element in the 3′-UTR of SPRY4-IT1. Moreover, SPRY4-IT1 was found to bind STAU1, promote STAU1 recruitment to the 3′-UTR of *TCEB1* mRNA, and affect *TCEB1* mRNA stability and expression, resulting in hypoxia-inducible factor 1α (HIF-1α) upregulation, and thereby affecting cancer cell metastasis. In addition, STAU1 depletion abrogated *TCEB1* SMD and alleviated the pro-metastatic effect of SPRY4-IT1 overexpression. Significantly, we revealed that SPRY4-IT1 is also transactivated by NF-κB/p65, which activates SPRY4-IT1 to inhibit *TCEB1* expression, and subsequently upregulate HIF-1α. In conclusion, our results highlight a novel mechanism of cytoplasmic lncRNA SPRY4-IT1 in which SPRY4-IT1 affecting *TCEB1* mRNA stability via STAU1-mediated degradation during cancer metastasis.

## Introduction

The metastatic spread of malignant cells to distant anatomical locations is a prominent cause of cancer-related death. Metastasis is a complex process, and many cell-intrinsic elements and extrinsic microenvironmental factors affect the metastatic potential of cancer cells [1]. However, the underlying molecular mechanisms that facilitate the metastatic cascade remain largely unclear. Thus, an enhanced understanding of such process might promote the development of effective metastasis-targeting therapy to improve the overall prognosis of patients with cancer.

Long noncoding RNAs (lncRNAs) are a class of transcripts longer than 200 nucleotides with limited protein coding potential [2]. Recently, many studies have shown that these are frequently deregulated in cancers and have multiple functions in a wide range of biological processes such as proliferation, apoptosis, and cell migration. Further, several lncRNAs such as HOTAIR [3], lncRNA-ATB [4], and PTAR [5], have been reported to modulate tumor metastasis. Whereas most characterized lncRNAs function in the nucleus, much less is known about their mode of action in the cytoplasm. A notable exception for this is competing endogenous RNAs, which act as molecular sponges for microRNAs to relieve the repression of target mRNAs [6, 7]. Other known mechanisms associated with lncRNAs in the cytoplasm involve post-transcriptional regulation, which affects mRNA stability or accessibility to the translational machinery [8, 9].

The RNA-binding protein Staufen1 (STAU1) is part of a highly conserved family of double-stranded RNA-binding proteins that have been implicated in mRNA transport, stability, and translation [10]. Further, it was previously shown to destabilize mRNAs in the cytoplasm [11] and bind a STAU1-binding site in the 3′-untranslated region (3′-UTR) of its target mRNA to induce mRNA degradation, which has been termed STAU1-mediated mRNA decay (SMD) [12]. This activity is also involved in developmental processes such as myogenesis and adipogenesis and is likely to be involved in angiogenesis [13–15]. However, its role in cancer is unclear. *TINCR*, a lncRNA that produces a 3.7-kb transcript, was first reported to bind STAU1 protein and mediate alterations to mRNA stabilization [16]. Cytoplasmic lncRNA, *SNHG5*, has been shown to control gene expression via SMD in colorectal cancer.

Here, we reported a novel role for SMD in cancer metastasis. We found that a cytoplasmic lncRNA, namely SPRY4 intronic transcript 1 (SPRY4-IT1), mediated cell metastasis by modulating *TCEB1* mRNA stability via SMD, which in turn increased HIF-1α expression and promoted the metastasis of cancer cells. Our results highlighted a novel molecular mechanism associated with the functions of a cytoplasmic lncRNA, via STAU1 activity, during cancer metastasis; this could thus comprise a potential therapeutic target for cancer.

## Results

### SPRY4-IT1 promotes metastasis in multiple types of human cancer cell lines and tumor tissues

**Fig. 1:**
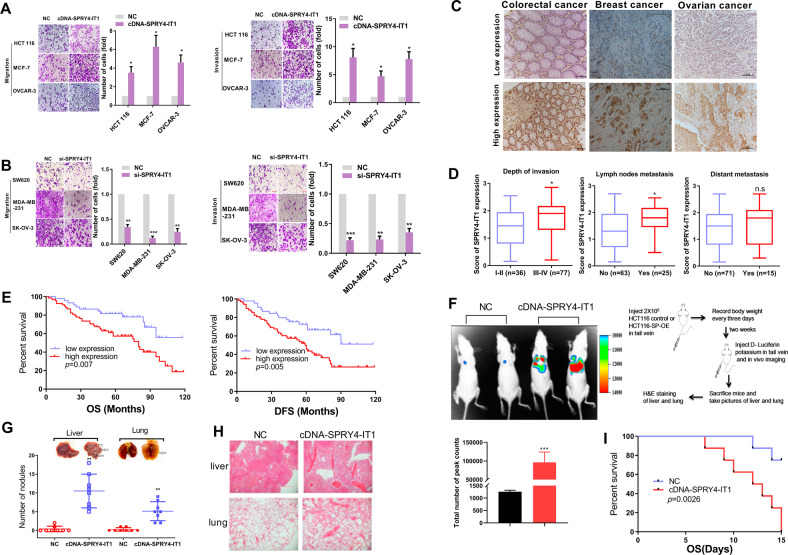
SPRY4-IT1 is associated with tumor aggressiveness. **A**, **B** Representative images and quantification of migration and invasion assays for HCT 116, MCF-7, and OVCAR-3 cells transfected with (**A**) SPRY4-IT1 cDNA or its control vector. SW620 were transfected with (**B**) siCon or siSPRY4-IT1 oligonucleotides. Numbers of migrated or invaded cells were counted from three replicate wells. The numbers in the control (Con) groups were set to 1. **C** Representative ISH images of SPRY4-IT1 in colorectal, breast, and ovarian cancer tissues. **D** Expression level of SPRY4-IT1 in colorectal cancer samples categorized by depth of invasion (advanced stage), lymph node metastasis, and distant metastasis. **E** Overall survival and disease-free survival analysis of colorectal cancer samples. The survival time of patients after surgery was compared between high-SPRY4-IT1 and low-SPRY4-IT1 expression groups. **F** Different clones were injected into the tail vein of male nude mice (*n* = 3). Bioluminescent signals were assayed 2 weeks after tail vein injection for Ctrl and SPRY4-IT1^OE^ HCT 116 stable cell lines. **G** Representative images of lungs and livers with metastatic nodules and statistical results. **H** HE staining of nude mouse liver and lung tissue. **I** Kaplan–Meier survival curve for different groups of mice.

Since SPRY4-IT1 is widely expressed in a variety of tumors, different cell lines were collected to determine whether it can broadly regulate tumor metastasis. We utilized a panel of cancer cells including colorectal breast, and ovarian cancer cell lines. The results confirmed that SPRY4-IT1 expression was positively correlated with invasion and migration in cancer cells (Supplementary Fig. S1a–c). Transwell assays revealed that the overexpression of SPRY4-IT1 in HCT 116, MCF-7, and OVCAR-3 cells promoted cancer cell migration and invasion (Fig. 1A, Supplementary Fig. S2a). In contrast, the knockdown of SPRY4-IT1 in SW620, MDA-MB-231, and SK-OV-3 cells significantly repressed cancer cell migration and invasion compared to those in control cells (Fig. 1B, Supplementary Fig. S2b).

To validate these findings, in situ hybridization assays were conducted to detect SPRY4-IT1 expression in 113 clinical colorectal cancer, 101 breast cancer, and 96 ovarian cancer tissue samples (Fig. 1C). We found that SPRY4-IT1 was significantly upregulated in colorectal cancer with lymph node metastasis and advanced-stage disease (Fig. 1D). Moreover, the Kaplan–Meier plot showed that both overall and disease-free survival were shorter in patients with high SPRY4-IT1 expression compared to that in the low SPRY4-IT1 expression subgroup of colorectal cancer patients (Fig. 1E). Similar results were also found in an independent cohort of breast cancer and ovarian cancer patients (Supplementary Fig. S3A, B).

In addition, we assessed the impact of SPRY4-IT1 on metastasis in vivo using the lung metastasis mouse model (Supplementary Information material: Stable cell line generations and In vivo experiments). These results revealed that SPRY4-IT1 overexpression significantly promotes colorectal cancer pulmonary and hepatic metastasis (Figs. 1F, 1G and 1H). More importantly, the overexpression of SPRY4-IT1 in HCT 116 cells also reduced mouse survival (Fig. 1I). Overall, our results demonstrated the ability of SPRY4-IT1, which was found to be upregulated in advanced colorectal, breast, and ovarian cancer tumor types, to promote cancer cell migration/invasion and metastasis, consistent with the previous report.

#### SPRY4-IT1 promotes TCEB1 mRNA decay by forming a duplex with 3′-UTRs via Alu elements

**Fig. 2:**
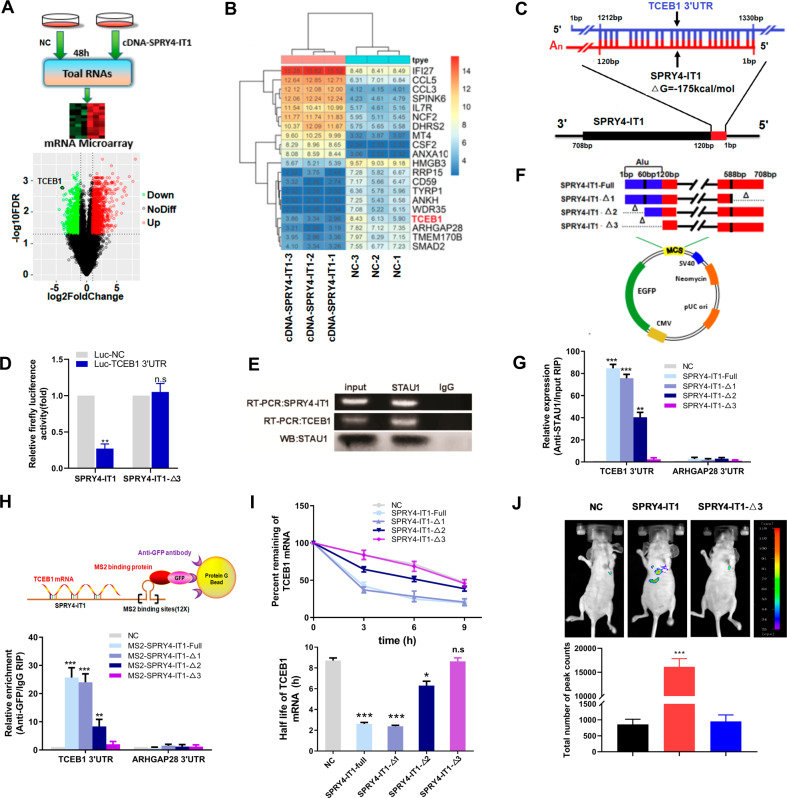
SPRY4-IT1 promotes *TCEB1* mRNA decay by forming duplexes with 3′-UTRs via Alu elements. **A** Upper: a schematic outline of the mRNA microarray analysis strategy used to identify SPRY4-IT1-associated mRNAs. Lower: volcano map indicating up- or downregulation of SPRY4-IT1-associated mRNAs. **B** Gene expression profiling in HCT 116 cells following SPRY4-IT1 overexpression. The heat map reveals clusters of the top 20 genes. **C** Predicted base-pairing between the Alu element in the 3′-UTR of *TCEB1* mRNA and the Alu element of SPRY4-IT1. **D** Luciferase reporter assay 48 h after transfection of 293 T cells with the indicated plasmids and a renilla luciferase transfection control plasmid. **E** RNA immunoprecipitation (RIP). IP was performed with a STAU1-specific antibody. Rabbit IgG was included as negative control for immunoprecipitation. The RNA was extracted and *TCEB1* mRNA and SPRY4-IT1 levels were evaluated. The RT-qPCR products were analyzed by RNA electrophoresis. **F** A schematic outline of the SPRY4-IT1 truncation strategy used to identify endogenous *TCEB1* mRNA–SPRY4-IT1 binding via Alu elements. **G** RIP was performed to determine the interaction between truncated SPRY4-IT1 variants and *TCEB1*. HCT 116 cells were transfected with full-length or SPRY4-IT1 truncations. *TCEB1* mRNA levels were evaluated by RT-qPCR. *ARHGAP28*, an mRNA without an Alu element was used as a negative control. **H** MS2-RIP followed by RT-qPCR to detect *TCEB1* endogenously associated with truncated SPRY4-IT1. **I** *TCEB1* mRNA stability in HCT 116 cells transfected with the indicated plasmids; 48 h after transfection, the cells were treated with triptolide at a final concentration of 10 mM at the indicated times and the RNA was subsequently extracted. Upper: the percentage of remaining *TCEB1* mRNA was obtained by normalizing to corresponding expression levels in the untreated cells. Lower: half-life of *TCEB1* mRNA. **J** The effect of SPRY4-IT1 mutant on CRC cell metastasis in vivo. Bioluminescent signals were assayed 2 weeks after tail vein injection for Ctrl, with full length SPRY4-IT1 (SPRY4-IT1-full) or mutant SPRY4-IT1 lacking 1-120 (SPRY4-IT1-Δ3) HCT 116 stable cell lines (*n* = 3). *p < 0.05, **p < 0.01, ***p < 0.001 vs. NC.

To elucidate the underlying mechanism through which SPRY4-IT1 exerts its pro-metastatic effects, global transcriptomes were analyzed in HCT 116 cells overexpressing SPRY4-IT1 (Supplementary Information material: microarray), and these were compared to those of negative control cells (Fig. 2A; triplicate repeats for each condition). Microarray analyses identified several genes that were significantly and differentially expressed after SPRY4-IT1 overexpression (Fig. 2B). Recently, cytoplasmic lncRNA has been shown to control gene expression via SMD in cancer. Given SPRY4-IT1 cytoplasmic location (Supplementary Fig. S4), we next focused on the role of SMD-associated mechanisms related to SPRY4-IT1 activity. Because SMD relies on base-pairing between the Alu element of a lncRNA and that of the 3′-UTR of mRNA, this led us to focus on mRNAs that contain a single 3′-UTR Alu element (Supplementary Table S4). Among the most significantly deregulated transcripts validated by qRT-PCR (Supplementary Fig. S5), the 3′-UTR of *TCEB1* mRNA was determined to have the potential to base-pair with the Alu element of the 3′-UTR of SPRY4-IT1 with ΔG values of −175 kcal/mol (Fig. 2C). We then assessed the binding of SPRY4-IT1 to the 3′-UTR of *TCEB1* using a luciferase reporter system. The ectopic expression of SPRY4-IT1 in 293 T cells increased the luciferase activity of the *TCEB1* 3′-UTR reporter construct, as compared with the control (Fig. 2D), suggesting that SPRY4-IT1 forms RNA–RNA interactions with *TCEB1* mRNA.

To validate the direct binding between SPRY4-IT1 and *TCEB1* via STAU1, we performed an anti-STAU1 RIP to pull-down endogenous lncRNAs and mRNAs associated with STAU1(Supplementary Information material: RIP). Interestingly, we demonstrated by RT-PCR analysis that the STAU1 immunoprecipitate from HCT 116 cells was significantly enriched in SPRY4-IT1 and *TCEB1* compared to that with IgG (Fig. 2E). To map the SPRY4-IT1 domain(s) required for *TCEB1* interactions, we generated various SPRY4-IT1 deletion mutants (Supplementary Fig. S6) and assessed their interactions with *TCEB1* by RIP using HEK293T cells (Fig. 2F). Based on this analysis, a SPRY4-IT1 deletion mutant (Δ2, Δ3) lacking the Alu element (1–120 bp) did not interact with STAU1, whereas all other mutants tested showed binding that was comparable to that of the wild-type protein (Fig. 2G). MS2-RIP was used to further validate the direct interaction between SPRY4-IT1 and *TCEB1*(Supplementary Information material: RIP). For this, the precipitated 3′-UTR of *TCEB1* was analyzed by real-time PCR. We found that MS2-tagged wild-type SPRY4-IT1 immunoprecipitate was enriched in the 3′-UTR of *TCEB1* compared to that with the empty vector and SPRY4-IT1 harboring a deletion (Δ2, Δ3) (Fig. 2H). We also overexpressed the panel of SPRY4-IT1 mutants and measured *TCEB1* mRNA stability following RNA PolII inhibitor Triptolide treatment. Interestingly, the SPRY4-IT1 mutant lacking the Alu element failed to promote *TCEB1* mRNA stability (Fig. 2I). We also found that STAU1 downregulation promoted TCEB1 mRNA stability in SPRY4-IT1-overexpressing HCT 116 cells (Supplementary Fig. S7). Importantly, SPRY4-IT1 deletion (Δ3) could not promote cancer metastasis in vitro (Supplementary Fig. S8) and in vivo (Fig. 2J).

To test the specificity of the SPRY4-IT1-TCEB1 mRNA interaction, we transfected two previously reported Alu-containing lncRNAs, FBXL19-AS1 and LINC00346 in HCT 116 cells, it was not observed for the binding of TCEB1 mRNA to STAU1 (Supplementary Fig. S9a). Also, overexpression of SPRY4-IT1 RNA was unable to cause other Alu-containing mRNAs (ANKH and RRP15) to bind to STAU1 (Shown in Supplementary Fig. S9b). These results suggested that SPRY4-IT1 interacts with *TCEB1* via STAU1.

#### The SPRY4-IT1–TCEB1 axis regulates metastasis in cancer cells

**Fig. 3:**
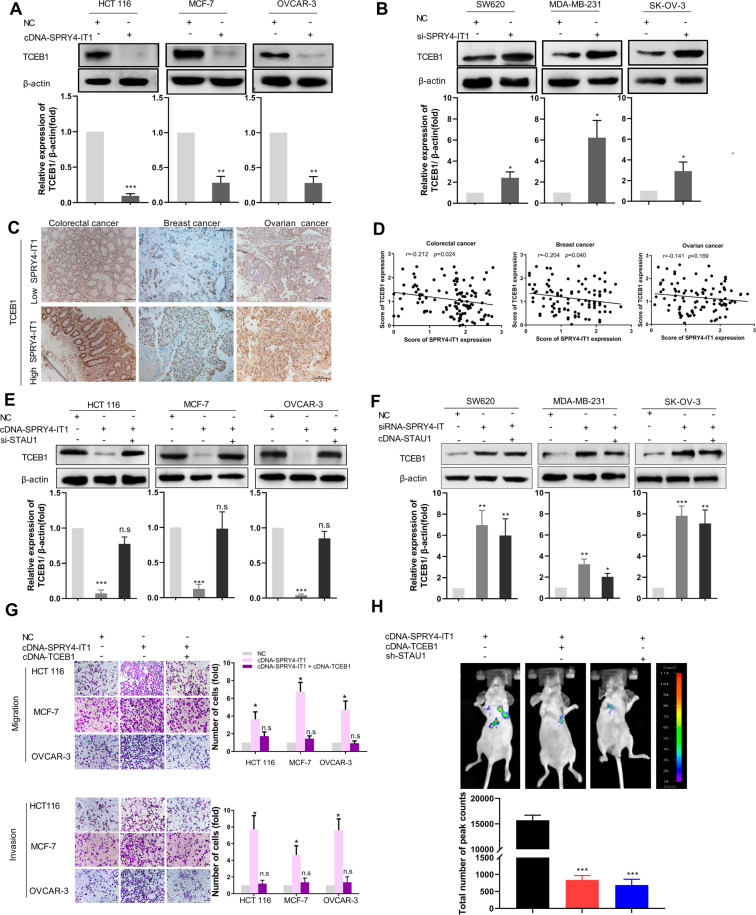
SPRY4-IT1 promotes the migration and invasion of human cancer cells through the negative regulation *TCEB1* and interactions with STAU1. **A** SPRY4-IT1 suppresses the protein expression of *TCEB1* in multiple cancer cell lines. Cell lysates from HCT 116, MCF-7, or OVCAR-3 cells with SPRY4-IT1 overexpression were subjected to western blot analysis. The immunoblots presented were derived from replicate samples run on parallel gels. **B** Similar experiments were performed in siSPRY4-IT1-expressing SW620, MDA-MB-231, and SK-OV-3 cells to study the functional roles of SPRY4-IT1. The immunoblots presented were derived from replicate samples run on parallel gels. **C** Representative colorectal, ovarian, and breast cancer tissues showing the expression of *TCEB1* in high- and low-SPRY4-IT1-expression tissue groups. Scale bars: 100 μm. **D** SPRY4-IT1 and TCEB1 expression scores were determined by analyzing their correlations in colorectal, ovarian, and breast cancers. **E**, **F** Cells were transfected with indicated siRNAs or cDNA for 48 h and analyzed for TCEB1 expression by western blot. **G** HCT 116, MCF-7, or OVCAR-3 cells overexpressing SPRY4-IT1 were transfected with control or TCEB1 cDNA for 48 h, and then cell migration and invasion were determined as described in the Materials and Methods. **H** SPRY4-IT1OE HCT 116 stably expressing SPRY4-IT1 plus TCEB1 cDNA or STAU1 shRNA were injected into the tail vein of nude mice (*n* = 3). The kinetics of cancer liver and lung metastasis was monitored and representative images are shown. *p < 0.05, **p < 0.01, ***p < 0.001 vs. NC.

Having confirmed the ability of SPRY4-IT1 to bind STAU1 and *TCEB1*, we next investigated whether this affected the expression of TCEB1. We ectopically overexpressed SPRY4-IT1 in HCT 116, MCF-7, and OVCAR-3 cells and observed significantly decreased levels of TCEB1 (Fig. 3A and Supplementary Fig. S10a), whereas knockdown of SPRY4-IT1 dramatically enhanced TCEB1 expression (Fig. 3B and Supplementary Fig. S10b). Moreover, we also found that higher levels of SPRY4-IT1 correlated with repressed TCEB1 protein levels in human colorectal, breast, and ovary cancer tissues (Figs. 3C and D), supporting the fact that SPRY4-IT1 mediates the down-regulation of TCEB1 in cancer.

Next, we determined whether TCEB1 expression, regulated by SPRY4-IT1, is mediated by STAU1; to address this, we administered si-STAU1 to MCF-7, HCT 116, and OVCAR-3 cells transiently overexpressing SPRY4-IT1. The silencing efficiency of si-STAU1 in cells was verified through qRT-PCR and western blot (Supplementary Fig. S11). The overexpression efficiency of cDNA-STAU1 was verified by western blot (Supplementary Fig. S12). As expected, the reduction in TCEB1 expression mediated by SPRY4-IT1 was almost completely reversed by the down-regulation of STAU1 (Fig. 3e and Supplementary Fig. S13a). The induction of TCEB1 expression by SPRY4-IT1 siRNA was not significantly influenced by the upregulation of STAU1 (Fig. 3F and Supplementary Fig. S13b). These results suggested that SPRY4-IT1 regulates TCEB1 expression in a STAU1-dependent manner.

Because our results showed that SPRY4-IT1 promotes cancer metastasis, we next addressed the functional role of TCEB1 in this phenotype. We ectopically overexpressed TCEB1 in SPRY4-IT1-overexpressing MCF-7, HCT 116, and OVCAR-3 cells and discovered that this could partially attenuate the increased cell migration and invasion mediated by SPRY4-IT1 overexpression (Fig. 3G and Supplementary Fig. S14). The decreased migration capacity associated with SPRY4-IT1 silencing in SW620, MDA-MB-231, and SK-OV-3 cells was also rescued by TCEB1 knockdown (Supplementary Fig. S15 and S16). To determine if SPRY4-IT1 dependent metastasis formation in vivo was mediated by TCEB1 and STAU1, we expressed TCEB1 cDNA in SPRY4-IT1-overexpressing HCT 116 cell line. As expected, TCEB1 overexpression partially reverted the prometastatic role of SPRY4-IT1 in HCT 116 in spontaneous metastasis assays. The overexpression of SPRY4-IT1 promoted pulmonic metastases in HCT 116 cells, which was also strongly abolished by inhibition of STAU1 (Fig. 3h). These results indicated that the mechanism through which SPRY4-IT1 functions in cancer cells might be attributed to SPRY4-IT1/TCEB1 complexes.

#### SPRY4-IT1-mediated suppression of TCEB1 activates HIF-1α signaling pathways

**Fig. 4:**
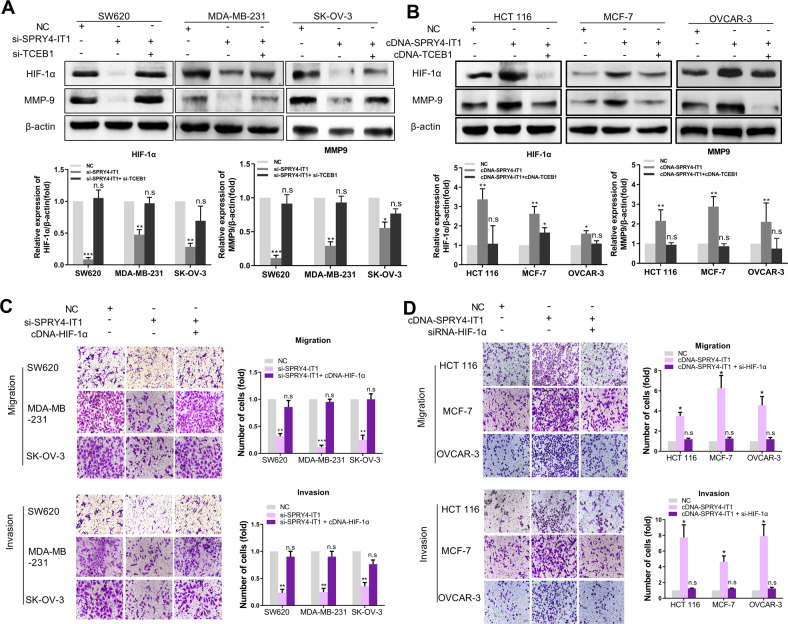
SPRY4-IT1 activates HIF-1α signaling pathways via TCEB1. **A** MDA-MB-231, SW620, and SK-OV-3 cells were transfected with NC, SPRY4-IT1 siRNA, or TCEB1 siRNA. The expression levels of HIF-1α and TCEB1 were analyzed by western blot. β-actin was used as a loading control. **B** MCF-7, HCT 116, and OVCAR-3 cells were transfected with SPRY4-IT1 cDNA (cDNA-SPRY4), TCEB1 cDNA (TCEB1), and the negative control (NC). The expression of HIF-1α and TCEB1 was analyzed by western blot. β-actin was used as a loading control. **C**, **D** Cell migration and invasion were detected by transwell assays. *p < 0.05, **p < 0.01, ***p < 0.001 vs. NC.

TCEB1 is a key factor for the formation and activity of E3 ubiquitin ligase complexes [17]. It has been reported that decreased TCEB1 expression or function can reduce the activity of E3 ubiquitin ligases and suppress the ubiquitination of hypoxia-inducible factor 1α (HIF-1α), resulting in enhanced expression [18]. We thus tested whether the SPRY4-IT1-mediated suppression of TCEB1 could activate HIF-1α signaling pathways. We found that the knockdown of SPRY4-IT1 in MDA-MB-231, SW620, and SK-OV-3 cells decreased HIF-1α and MMP-9 protein expression, whereas TCEB1 inhibition rescued this effect (Fig. 4A). Inversely, overexpression of SPRY4-IT1 in HCT 116, MCF-7, and OVCAR-3 cells could increase HIF-1α and its downstream factor MMP-9, whereas TCEB1 overexpression abrogated SPRY4-IT1-induced HIF-1α and MMP-9 upregulation (Fig. 4B). However, mRNA levels of *HIF-1α* were not significantly changed upon SPRY4-IT1 manipulation (Supplementary Fig. S17).

We then investigated whether SPRY4-IT1 regulates migration and invasion by modulating HIF-1α. The overexpression of HIF-1α in MDA-MB-231, SW620, and SK-OV-3 cells rescued the diminished migration and invasion ability induced by SPRY4-IT1 knockdown (Fig. 4C and Supplementary Fig. S18). Conversely, in HCT 116, MCF-7, and OVCAR-3 cells, the overexpression of promoting effect of SPRY4-IT1 on migration and invasion was dampened with HIF-1α knockdown (Fig. 4D and Supplementary Fig. S19). These data suggest that HIF-1α might be the downstream effector of SPRY4-IT1 during cancer metastasis.

#### NF-κB is an upstream regulator of SPRY4-IT1

**Fig. 5:**
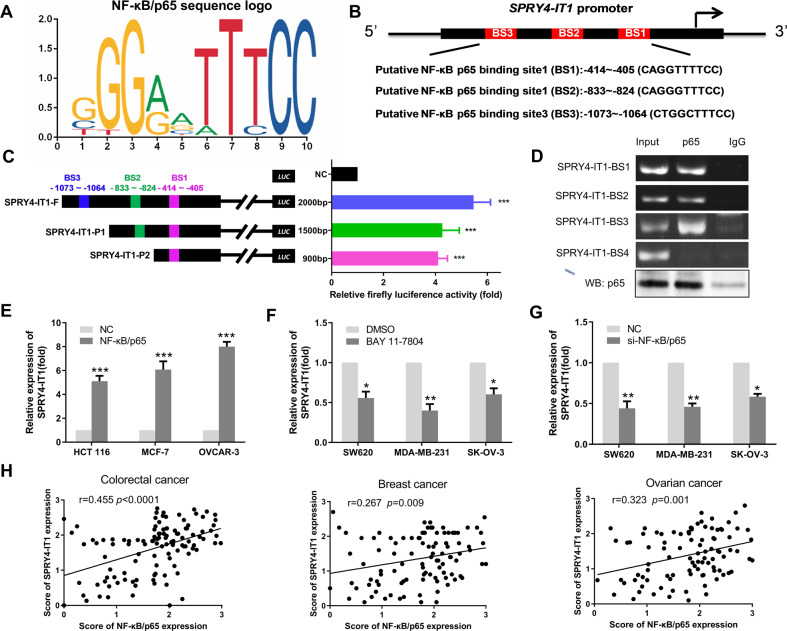
NF-κB binds the promoter regions of SPRY4-IT1 and transcriptionally upregulates its expression. **A** The sequence logo of NF-κB/p65 was predicted by the JASPAR database. **B** Schematic of predicted binding sites (BS) between SPRY4-IT1 and NF-κB/p65. **C** Construction of luciferase reporter vectors comprising different truncation variants of SPRY4-IT1. Dual-luciferase reporter assays were performed by co-transfecting the full-length SPRY4-IT1 promoter (SPRY4-F) or truncated SPRY4-IT1 (SPRY-P1, SPRY4-P2) with NF-κB/p65 cDNA plasmid or empty vectors in 293 T cells. Relative luciferase activity was then assayed. **D** ChIP assay showing endogenous NF-κB/p65 bound to the SPRY4-IT1 promoter in HCT 116 cells. **E** RT-qPCR analysis of SPRY4-IT1 expression in MCF-7, HCT 116, and OVCAR-3 cells following the transient transfection of NF-κB/p65 cDNA. **F**, **G** RT-qPCR analysis of SPRY4-IT1 expression in MDA-MB-231, SK-OV-3, and SW620 cells treated with an NF-κB inhibitor (**F**) and siRNA (**G**) for 48 h. **H** SPRY4-IT1 and NF-κB/p65 expression scores were determined by analyzing correlations between these markers in colorectal, ovarian, and breast cancers.

Increasing evidence indicates that transcription factors such as E2F1 and SP1 can activate the transcription of downstream targets including lncRNAs [19, 20]. To gain further insight into the regulation of SPRY4-IT1 transcription, we utilized the JASPAR (http://jaspar.genereg.net/) online prediction tool to identify potential transcription factors that regulate SPRY4-IT1 expression. NF-κB/p65 was found to bind the SPRY4-IT1 promoter region with a high score (Figs. 5A and 5B). Next, we characterized the binding of NF-κB/p65 to the promoter region of SPRY4-IT1. Subsequent luciferase reporter assays using a group of SPRY4-IT1 promoter constructs confirmed the functional involvement of these NF-κB-binding motifs (Supplementary Information material: Luciferase activity assay). Compared to that with the negative control, the pCMV-NF-κB/p65 plasmid remarkably enhanced luciferase activity from SPRY4-IT1 reporters in 293 T cells (Fig. 5C). ChIP-PCR assays (Supplementary Information material: CHIP) also indicated that each of these binding sites was indeed a bona fide motif that was bound by NF-κB/p65 (Fig. 5D). We also found that the transfection of HCT 116, MCF-7, and OVCAR-3 cells with a NF-κB/p65 expression vector could increase SPRY4-IT1 expression (Fig. 5e and Supplementary Fig. S20). Conversely, the specific NF-κB inhibitor BAY 11-7802 and siRNA targeting NF-κB (si-NF-κB/p65) suppressed SPRY4-IT1 expression (Figs. 5F, G and Supplementary Fig. S21). However, levels of SPRY4-IT1 were not significantly changed upon NF-κB/p50 manipulation (Supplementary Fig. S22). NF-κB and SPRY4-IT1 levels were also positively correlated in colorectal, breast, and ovarian cancer tissues (Fig. 5H, *p* < 0.05). Taken together, these findings provided compelling evidence that SPRY4-IT1 is a direct transcriptional target of NF-κB/p65.

#### NF-κB promotes metastasis by activating SPRY4-IT1 transcription and regulates TCEB1 and HIF-1α

**Fig. 6:**
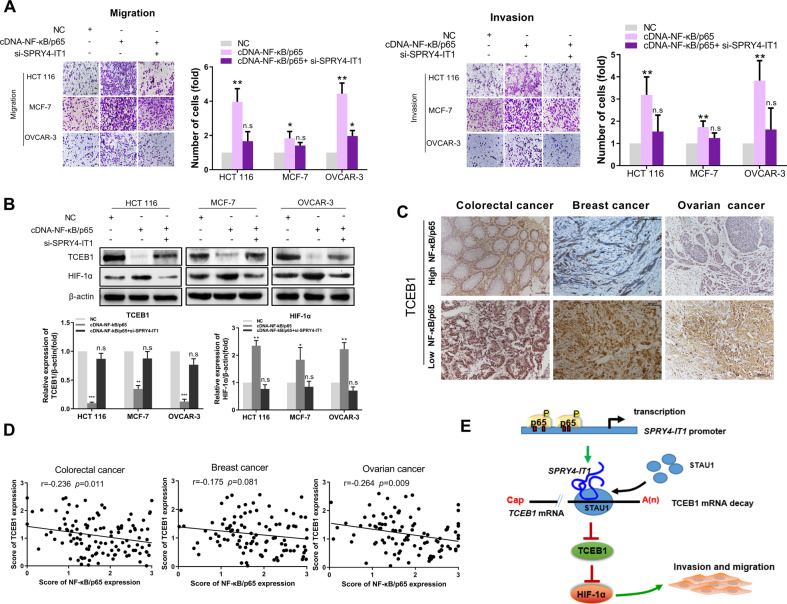
NF-κB promotes metastasis by activating SPRY4-IT1 transcription and regulating TCEB1 and HIF-1α. **A** MCF-7, HCT 116, and OVCAR-3 cells were transfected with NF-κB/p65 cDNA plus siSPRY4-IT1, and cell migration and invasion were measured by transwell assays. **B** The expression of HIF-1α and TCEB1 were analyzed by western blot. Cell lines were treated as in (**A**). **C** Representative colorectal, ovarian, and breast cancer tissues showing the expression of TCEB1 in high- and low-NF-κB/p65 expression tissue groups. Scale bars: 100 μm. **D** Negative relationship between TCEB1 and NF-κB/p65 expression in colorectal, ovarian, and breast cancer tissues. *p < 0.05, **p < 0.01, ***p < 0.001 vs. NC. **E** Proposed model in which SPRY4-IT1 mediates the migration and progression of cancer cells.

In cancer cells, the NF-κB family of transcription factors plays pivotal role in both promoting and maintaining an invasive phenotype [21]. We, therefore, interrogated the role of SPRY4-IT1 in mediating cancer cell migration as it relates to NF-κB. As expected, transfecting HCT 116, MCF-7, and OVCAR-3 cells with NF-κB/p65-encoding cDNA increased migration and invasion, which was partially abolished upon SPRY4-IT1 silencing (Fig. 6A). Further, we ectopically expressed NF-κB/p65 in HCT 116, MCF-7, and OVCAR-3 cells and analyzed TCEB1 and HIF-1α levels. Results showed that the introduction of exogenous NF-κB expression could efficiently downregulate TCEB1 and increase HIF-1α expression in cancer cells. Consistently, the ectopic expression of SPRY4-IT1 abolished these effects (Fig. 6B). We also found that NF-κB/p65 and TCEB1 were negatively correlated in colorectal, breast, and ovarian cancer tissues (*p* < 0.05, Fig. 6C, D). These observations are consistent with our model and support the notion that NF-κB activates SPRY4-IT1 to inhibit TCEB1 expression and subsequently increase HIF-1α expression.

## Discussion

In this study, we provided insight into the molecular mechanisms underlying cancer metastasis by identifying a new pathway that affects this process. Specifically, SPRY4-IT1 was found to have an oncogenic function with respect to cancer metastasis in part by promoting STAU1-mediated *TCEB1* mRNA decay; this was achieved through the formation of duplexes with 3′-UTRs via Alu elements, and subsequently the upregulation of the HIF-1α signaling pathway. Importantly, NF-κB was found to bind directly to the SPRY4-IT1 promoter region to activate transcription and ultimately inhibit TCEB1 expression, subsequently increasing HIF-1α expression. A model summarizing these concepts is presented in Fig. 6e.

SPRY4-IT1, which is located on chromosome 5q31.3 and derived from an intron within the *SPRY4* gene, was first identified as an oncogene in melanoma, as it plays an important role in melanoma cell growth, apoptosis, migration, and invasion [22].

Providing new insight into the role of SPRY4-IT1 in cancer was an important outcome of this study because its precise functions have been difficult to resolve, with reports of both tumor-suppressive and oncogenic activity. For example, SPRY4-IT1 is upregulated in many cancer types including breast cancer, cholangiocarcinoma [23], pancreatic ductal adenocarcinoma [24], hepatic cellular carcinoma [25, 26], and melanoma [22]. In contrast, it appears to be downregulated in non-small-cell lung cancer [27] and gastric cancer [28] and acts as a potential tumor suppressor. This suggests that this lncRNA might exert an oncogenic or tumor suppressor function depending on the tissue-specific expression pattern and context. Our demonstration that SPRY4-IT1 promotes invasion, migration, and metastasis is consistent with the former findings. In our study, to ensure that findings were not predicated on a single model and therefore likely to be robust, we utilized multiple cell lines (including colorectal, breast, and ovarian cancer) for in vitro assays, as well as different tissues. Promisingly, SPRY4-IT1 induced metastasis not only in colorectal cancer cells but also in ovarian and breast cancer cells, indicating that it is a more general promoter of metastasis.

Currently, the mechanism underlying the function of SPRY4-IT1 in tumors is not well-defined. SPRY4-IT1 is known to have both nuclear and cytoplasmic versions. In the nucleus, SPRY4-IT1 domains interact with the intronic regions of *SMYD3*, *SND1*, *MEOX2*, *SOX5*, *RASAL2*, and *DCTN6* pre-mRNAs [29]. In the cytoplasm, SPRY4-IT1 by functioning as ceRNAs or “RNA sponges” regulating *AMPK, EZH2* mRNA stability [23, 30]. In the present study, we report that SPRY4-IT1 is localized preferentially in the cytoplasm, as determined by FISH experiments; We identified the involvement of SMD in SPRY4-IT1-mediated cancer metastasis. In 2011, a novel regulatory mechanism was identified in which lncRNAs transactivate SMD by forming duplexes with 3′-UTRs via Alu elements [31]. In such cases, STAU1-binding sites can be formed by imperfect base-pairing between an Alu element in the 3′-UTR of an SMD-target and another Alu element in a cytoplasmic, polyadenylated lncRNA. Damas and colleagues showed that lncRNA-SNHG5 promotes colorectal cancer cell survival by counteracting STAU1-mediated *SPATS2* mRNA destabilization [32]. Yang et al. showed that LINC00346 can bind to STAU1 and promote the degradation of ZNF655 mRNA [33]. Given its cytoplasmic location, the SPRY4-IT1-mediated control of genes might occur at the post-transcriptional level through direct associations with target mRNAs. To determine the SMD-associated mechanisms related to SPRY4-IT1 activity, we performed RNA transcriptome sequencing to identify differentially expressed genes between control and SPRY4-IT1-overexpressing cells. Studies showed that STAU1-binding sites can be created by imperfect base pairing between an Alu element of an mRNA target of SMD and another Alu sequence in a half-STAU1-binding site lncRNA. Therefore, we focused on mRNAs that contain a single 3′-UTR Alu-element. We showed that *TCEB1* is sensitive to STAU1-mediated mRNA decay and that SPRY4-IT1 can promote the association between STAU1 and TCEB1. We also observed that STAU1 knockdown could rescue the metastatic phenotype mediated by SPRY4-IT1 overexpression, thus confirming the genetic interaction between the SPRY4-IT1 and STAU1, which promotes the degradation of *TCEB1*. Furthermore, TCEB1 functions as a key factor in E3 ubiquitin ligase complexes and can regulate HIF-1α expression [18]. HIF-1α regulates different genes to facilitate cancer metastasis [34, 35]. Notably, this protein was found to be remarkably upregulated upon SPRY4-IT1 overexpression. Thus, based on our results here, TCEB1 and HIF-1α could be crucial SPRY4-IT1 targets. Furthermore, our data demonstrated that the interaction between SPRY4-IT1 and STAU1 is required for the aforementioned functions in cancer.

HIF-1α is one of the best-studied oncogenes, as it transcriptionally regulates genes that facilitate cancer metastasis. Further, TCEB1 is a key factor in the formation and activity of E3 ubiquitin ligase complexes [36, 37]. It has been reported that decreased TCEB1 expression or function can suppress the ubiquitination of HIF-1α, enhancing its expression, and mediating the tumorigenesis of clear-cell renal cell carcinoma [18]; however, this is not well-understood. In this study, we found that SPRY4-IT1 upregulates HIF-1α protein levels in a TCEB1-dependent manner. Thus, SPRY4-IT1 comprises an additional layer of HIF-1α regulation in cancer cells. Whereas Lys391 and Lys477 of HIF-1α are the major ubiquitination sites, it remains unclear which is regulated by SPRY4-IT1 in a TCEB1-dependent manner. These questions should be addressed by future experiments, which could shed further light on the role of SPRY4-IT1 in tumor metastasis and its associated mechanism.

NF-κB is constitutively active in many tumors and considered a key factor for cancer development. In this study, our data demonstrated that SPRY4-IT1 overexpression in cancer cells could be activated by NF-κB, which can also promote the expression of the lncRNA NKILA [38]. We also showed that SPRY4-IT1 mediates the promoting effect of NF-κB on tumor metastasis [39]. Some studies suggested that NF-κB is involved in cancer progression; conventional explanations for this phenomenon include the induction of the expression of proto-oncogenes such as *c-myc* [40] and *cyclin D1* [41], adhesion molecules such as VEGFs [42] and MMPs [43], and miRNAs [44, 45]. Our study identified a new mechanism through which NF-κB activates SPRY4-IT1, which consequently inhibits TECB1 and activates HIF-1α signaling. Thus, the NF-κB/SPRY4-IT1/TCEB1/HIF-1α axis might play a critical role in facilitating metastatic processes.

In summary, we explored a novel mechanism through which SPRY4-IT1 induces the degradation of target mRNAs, and found that the binding between *TCEB1* and SPRY4-IT1 is dependent on STAU1 and that the induction of target mRNA degradation is an important function of STAU1. Therefore, the promotion of mRNA degradation might serve as an important mechanism underlying SPRY4-IT1 functions. Our results ultimately indicated that a strategy resulting in the destabilization of binding between SPRY4-IT1 and STAU1, using a specific agent such as a small molecule, could be employed to treat tumor patients harboring metastases.

## Materials and methods

Additional detailed Materials and Methods are available in the Supplementary Information (SI). The sequences of probe and primer are shown in Supplementary Table S1. The information of antibodies is shown in Supplementary Table S2. The information of siRNAs or shRNA is shown in Supplementary Table S3.

### Cell culture and reagents

The human colorectal HCT 116 (CVCL_0291), Caco-2(CVCL_0025), HT-29 (CVCL_0320), SW480 (CVCL_0546), SW620 (CVCL_0547), breast MCF-7(CVCL_0031), T-47D (CVCL_0553), MDA-MB-231(CVCL_0062), ovarian Caov-3(CVCL_0231), SK-OV-3(CVCL_0532) cancer cell lines and HEK293T(CVCL_0063) were obtained from the GeneChem Corporation (Shanghai, China). OVCAR-3(CVCL_0465) was obtained from the Nanjing KeyGen Biology (Nanjing, China). All human cell lines have been authenticated using STR profiling. OVCAR-3, Caco-2, MCF-7, Caov-3 and HEK293T cells were cultured in DMEM (Gibco, Carlsbad, CA, USA). T-47D and OVCAR-3 cells were cultured with RPMI 1640 (Gibco, Carlsbad, CA, USA). HCT 116 and HT-29 cells were cultured with McCoy’s 5a (Gibco, Carlsbad, CA, USA). SW480, SW620, and MDA-MB-231 cells were cultured with Leibovitz’s L-15 (Gibco, Carlsbad, CA, USA) medium containing 10% fetal bovine serum, 100 U/mL penicillin, and 100 mg/mL streptomycin. To inhibit NF-κB activities, BAY 117082 (Selleckchem, Houston, TX, USA) was used.

### Patients tissue samples

Formalin-fixed, paraffin-embedded samples (113 colorectal, 101 breast, and 96 ovarian cancer) were obtained from the First Affiliated Hospital and Shengjing Hospital of China Medical University, between 2009 and 2012. None of the patients had received radiotherapy or chemotherapy before surgery. Informed consent was obtained from each patient. Processing of patient material and data has been approved by the ethics committee of China Medical University (reference number: [2016]054).

### Statistical analyses

Statistical analyses were performed with SPSS 19.0 (IBM, SPSS, Chicago, IL, USA) and GraphPad Prism 5.0 (GraphPad Software Inc., CA, USA). The data were expressed as means ± standard deviation (SD) and were analyzed by either a Student *t* test (two samples) or one-way analysis of variance (ANOVA) when the variance is similar between the groups. The survival rates were evaluated by the Kaplan–Meier method and the log-rank test. Correlations between groups were analyzed by Pearson correlation analysis. Results with P values less than 0.05 were considered statistically significant.

The sample size for cell experiments, usually three or more, is based on the size and consistency of measurable differences between groups. No statistical method was used to predetermine the sample size for xenograft mice experiment, which was based on previous experimental observations. The sample size of each experiment is shown in the legend. No data were excluded from the analysis.

### Experimental procedures

Cancer cell lines were cultured and transfected with either SPRY4-IT1 siRNA or an overexpression vector. The metastatic capacity of the cells was assessed by transwell migration/invasion assays in vitro and by mouse tail–vein injection assays in vivo. To elucidate the SMD-associated mechanism through which SPRY4-IT1 exerts its pro-metastatic effects, mRNA microarray and bioinformatics prediction were used to identify the 3′-UTR of mRNAs that contain Alu elements. RNA immunoprecipitation assays (RIP) were carried out to verify the interaction between STAU1 and its binding RNAs. Further, the JASPAR CORE database was used to predict the transcriptional regulator of SPRY4-IT1. A luciferase reporter system, chromatin immunoprecipitation (ChIP) assays, and immunoblotting assays were used to investigate the regulatory targets and interacting proteins of SPRY4-IT1. Rescue experiments were performed to exclude the possible off-target for RNA knockdown experiments using re-expression of the same gene. Towards this, cDNA version was used. Clinical colorectal, breast, and ovarian patient tumor samples were used to study all clinical significance.

## Supplementary information

Supplementary Information

Supplementary Table S1

Supplementary Table S2

Supplementary Table S3

Supplementary Table S4

Supplementary Figure S1

Supplementary Figure S2

Supplementary Figure S3

Supplementary Figure S4

Supplementary Figure S5

Supplementary Figure S6

Supplementary Figure S7

Supplementary Figure S8

Supplementary Figure S9

Supplementary Figure S10

Supplementary Figure S11

Supplementary Figure S12

Supplementary Figure S13

Supplementary Figure S14

Supplementary Figure S15

Supplementary Figure S16

Supplementary Figure S17

Supplementary Figure S18

Supplementary Figure S19

Supplementary Figure S20

Supplementary Figure S21

Supplementary Figure S22

## Data Availability

The data that support the findings of this study are available from the corresponding author upon reasonable request. Microarray data are available at Gene Expression Omnibus with accession number GSE140616.
