# Supplementary material for: NF-κB-activated SPRY4-IT1 promotes cancer cell metastasis by downregulating TCEB1 mRNA via Staufen1-mediated mRNA decay
Source: Oncogene. 2021 Jun 23;40(30):4919–29. doi: 10.1038/s41388-021-01900-8 (PMC8321898; doi:10.1038/s41388-021-01900-8)
Supplement: Supplementary file 1 — Supplementary Information [file 41388_2021_1900_MOESM1_ESM.docx]

**NF-κB-activated SPRY4-IT1 promotes cancer cell metastasis by down-regulating TCEB1 mRNA via Staufen1-mediated mRNA decay**

Lin Zhao^1,2┼^, Longyang Jiang^1,2┼^, Ming Zhang^1,2^, Qiang Zhang^1,2^, Qiutong Guan^1,2^, Yalun Li^3^, Miao He^1,2^, Jingdong Zhang^4*^，Minjie Wei^1,2*^

**Supplementary Methods and Materials.**

**Quantitative PCR and Western blot analysis**

Quantitative PCR and Western blot analysis assays were performed as described in our previous studies [[1](#_ENREF_1), [2](#_ENREF_2)]. Specifically, the exposure time of HIF-1α was extended to 5 minutes due to the low basic expression of HIF-1α under normal oxygen conditions. The specific primers and antibodies are listed in Table S1 and 2.

**Oligonucleotide, plasmid transfection**

Effective siRNA oligonucleotides and cDNA of SPRY4-IT1 were purchased from RiboBio (RIBOBIO Technology Co., Ltd. Guangzhou, China). Effective siRNA/shRNA of TCEB1, STAU1, NF-κB/p65 or HIF-1α were purchased from Santa cruz (Santa Cruz Biotechnology, USA). To exclude of target effects of siRNA, scrambled sequence was used as a negative control. In siRNA experiments, both mRNA and protein levels were monitored to validate the RNAi target. The siRNA and cDNA were transfected with Lipofectamine 2000 (Invitrogen) according to the manufacturer’s instructions. The information of siRNAs is listed in Table S1 and 4.

**Stable cell line generations**

For lentiviral preparation, overexpression of SPRY4-IT1 in HCT 116 cells were achieved using the GV367 lentiviral vector (Genechem, Shanghai, China). The vector was packaged in 293 cells. Recombinant lentiviruses were produced by transient transfection of HEK293T cell. The supernatant fraction containing lentiviral particles was collected at 72 hr, followed by infection into HCT 116 cells supplemented with 10 μg/ml polybrene. At 12 hr after infection, the medium was replaced with fresh medium containing 0.5 mg/mL puromycin until all control cells (those not infected) died (usually 2–3 days) in the puromycin medium. All the plasmids were confirmed by DNA sequencing.

**RNA immunoprecipitation (RIP) assay**

RIP were performed as previously reported [[3](#_ENREF_3)].HCT 116 cells were transfected with pcDNA-SPRY4-IT1, pcDNA-SPRY4-IT1-truncations. After 48 hours, cells were used to perform RIP using the STAU1 antibody and Magna RIP RNA-binding protein Immunoprecipitation Kit (Millipore) according to the manufacturer’s instructions. Then the expression of TCEB1 mRNA was analyzed by qPCR. For MS2bs-MS2bp–based RIP assay, HCT 116 cells were transfected with pcDNA-SPRY4-IT1-MS2, pcDNA-SPRY4-IT1-truncations-MS2. After 48 hours, cells were used to perform RIP using a Magna RIP RNA-Binding Protein Immunoprecipitation Kit (Millipore). Then the expression of TCEB1 mRNA was analyzed by qPCR.

**Luciferase activity assay**

The assays were performed as previously described in our published paper [[2](#_ENREF_2)]. To figure out the direct binding site of NF-κB/p65 to the promoter region of SPRY4-IT1, the SPRY4-IT-promoter reporter constructs were co-transfected with full length NF-κB cDNA (NF-κB-F), or truncated SPRY4-IT1(SPRY-P1, SPRY4-P2) into HEK-293T cells using Lipofectamine 2000. After transfection for 48 h, luciferase activity was detected using the Dual Luciferase Reporter Gene Assay kit (Promega). Relative luciferase activity was normalized to the control.

**Chromatin immunoprecipitation (ChIP) assay**

ChIP assay was performed with a commercially available kit (Millipore, Merck KGaA, Darmstadt, Germany, 17-295), according to the manufacturer’s instructions. Briefly, cells (2×10^6^) were crosslinked in 1% formaldehyde for 10 min at room temperature, then the crosslink was stopped with 0.125 M glycine. Cells were sonicated and the collected supernatant was incubated with dyna beads protein G and 4μg of NF-κB/p65 primary antibody (Millipore, Merck KGaA, Darmstadt, Germany, 17-10060, mouse monoclonal) at 4°C, IgG was used as negative control. After 12h, chromatin was collected, purified, and de-crosslinked at 65°C. The precipitated DNA fragments were quantified by PCR analysis, using the primers shown in Supplementary Table S1.

**mRNA microarray**

The mRNA microarray profiling was carried out using the Agilent Whole Genome Oligo Microarrays (Cat.no. G4112F, 4×44k arrays). The quantification and quality check of total RNA were performed through NanoDrop ND-1000 Spectrophotometer (Thermo Scientific, Waltham, MA). RNA labeling, microarray hybridization, array scanning, data extraction and result analysis were performed by the KangChen Bio-tech company (Shanghai, China). The threshold value used to screen differentially expressed mRNAs was a fold change of ≥2.0 or ≤0.5, a P-value of less than 0.01, and a normalized signal value, indicating the relative abundance to the transcript, of ≥2.0. Microarray data are available at Gene Expression Omnibus with accession number GSE 140616. The whole datasets have been deposited in the Gene Expression Omnibus database (GSE140616).

**Histology and Immunohistochemistry (IHC)**

Fixed tissue was paraffin-embedded, sectioned (5-μm), deparaffinized in xylene, and rehydrated in graded ethanol. Samples were stained with hematoxylin and eosin (H&E) or the indicated antibodies (Supplementary Information Table S2.) according to standard protocol. Both the intensity and extent of immunoreactivity were evaluated and scored. IHC staining and score evaluation was performed according to standard protocols as described previously in our published article [[4](#_ENREF_4)].

**In situ hybridization (ISH)**

The relative expression of SPRY4-IT1 in tumor tissues was detected by in situ hybridization with a specific digoxin-labeled SPRY4-IT1 probe (5’-Dig- AATTTATGTGGCTGACAAAGGA-Dig-3’) (BOSTER, Boster Biological Technology Co., Ltd. Wuhan, China). Concisely, after dewaxed in xylene and rehydrated through gradient alcohol, the slides were digested by proteinase K 10 μg/ml at 37°C for 10min and hybridized with the specific SPRY4-IT1 probe at 4 °C overnight, then incubated with anti-Digoxin-AP (BOSTER) at 4 °C overnight. The tissues were stained with DAB (BOSTER) and quantified as described previously in our published article [4].

**Fluorescence** **in situ hybridization (FISH)**

The cells were washed by PBS for 5 minutes three times, and then fixed in 4% paraformaldehyde for 10 minutes. The cells were washed by PBS for 5 minutes three times, and then permeabilized using 0.5% Triton X-100 for 5 minutes and washed by PBS for 5 minutes three times. Hybridization with SPRY4-IT1 probes was performed at 37℃ overnight, followed by incubation with DAPI for 10 minutes (5’-Cy3- AATTTATGTGGCTGACAAAGGA-Cy3-3’) (RIBOBIO Technology Co., Ltd. Guangzhou, China). The cells were imaged using a confocal laser scanning microscope (NIKON，Japan).

**Transwell migration/invasion assays**

The ability of the cells to migrate and invade was assessed using Corning Transwell insert chambers with pores 8 μm in size (Corning) and a BD BioCoat Matrigel Invasion Chamber (Becton Dickinson Biosciences), respectively. Transfected cells in 200 μl of serum-free medium were seeded in the upper well; the chambers were then incubated with 20% fetal bovine serum medium for 48 h at 37°C to allow the cells to migrate to the lower well. The cells that had migrated or invaded through the membrane were fixed in methanol, stained with crystal violet (Invitrogen), imaged and counted.

***In vivo* experiments**

Before tumor cell inoculation mice were randomized into different groups with approximately equivalent numbers. The investigator was blinded to the group allocation of the animals during the experiment. HCT 116 cells was transfected with pGKV5 luciferase and then treated with G418 to obtain a cell line with luciferase expressing stably. The stable cell line was then treated with control or SPRY4-IT1 cDNA to generate two groups for athymic BALB/C mice (4 weeks old, female, Chinese Academy of Sciences, Shanghai, China) tail-vein injection. Each mouse was injected with 2×10^6^ cells in 100μl PBS. Bioluminescence imaging was used to detect the metastatic foci weekly. Mice were injected with D-Luciferin (300 mg/kg, 5 min prior to imaging), anesthetized with 3% isoflurane, and then imaged in an In-Vivo Imaging System (Carestream Molecular Imaging, USA). Each tissue was excised and embedded in paraffin for histopathological examination.

**Supplementary Figures**

**Supplementary References**

1 Zhao L, Wang Y, Jiang L, He M, Bai X, Yu L *et al*. MiR-302a/b/c/d cooperatively sensitizes breast cancer cells to adriamycin via suppressing P-glycoprotein(P-gp) by targeting MAP/ERK kinase kinase 1 (MEKK1). *Journal of experimental & clinical cancer research : CR* 2016; 35: 25.

2 Bi J, Zeng X, Zhao L, Wei Q, Yu L, Wang X *et al*. miR-181a Induces Macrophage Polarized to M2 Phenotype and Promotes M2 Macrophage-mediated Tumor Cell Metastasis by Targeting KLF6 and C/EBPalpha. *Molecular therapy Nucleic acids* 2016; 5: e368.

3 Gong C, Maquat LE. lncRNAs transactivate STAU1-mediated mRNA decay by duplexing with 3' UTRs via Alu elements. *Nature* 2011; 470: 284-288.

4 Wu H, Wang H, Guan S, Zhang J, Chen Q, Wang X *et al*. Cell-specific regulation of proliferation by Ano1/TMEM16A in breast cancer with different ER, PR, and HER2 status. *Oncotarget* 2017; 8: 84996-85013.

**Legends**

**Supplementary Table S1.** The sequences for primers used in the study.

**Supplementary Table S2.** Antibodies used for IHC, RIP, CHIP and WB.

**Supplementary Table S3.** Details of pairing between SPRY4-IT1 and top 10 down regulated mRNA.

**Supplementary Table S4.** The siRNA or shRNA used for silencing target genes.

**Supplementary Figure S1.** Expression levels of SPRY4-IT1 in multiple cancer cell lines. qRT-PCR analysis of SPRY4-IT1 expression in colorectal (HCT 116, Caco-2, HT-29, SW480, SW620), breast (MCF-7, T-47D, MDA-MB-231) and ovarian (OVCAR-3, Caov-3, SK-OV-3) cancer cell lines. *p<0.05, ** p<0.01, ***p<0.001 vs. low invasion and migration ability cells (HCT 116, MCF-7 or OVCAR-3).

**Supplementary Figure S2.** Exogenous regulation of SPRY4-IT1 by siRNA or cDNA in colorectal, breast and ovarian cancer cell lines. (a) Cells were transfected with siRNA-SPRY4-IT1 (10 nM) or control siRNA for 48h and expression levels of SPRY4-IT1 were determined by qRT-PCR analyses. (b) Cells were transfected with cDNA-SPRY4-IT1 (20 nM) or control cDNA for 48h and expression levels of SPRY4-IT1 were determined by qRT-PCR analyses. (c) 293T Cells were transfected with cDNA-SPRY4-IT1-△1/△2/△3 (20 nM) or control cDNA for 48h and expression levels of SPRY4-IT1-△1/△2/△3 were determined by qRT-PCR analyses. *p<0.05, ** p<0.01, *** p<0.001 vs. control group.

**Supplementary Figure S3.** SPRY4-IT1 correlates with poor prognosis in breast cancer and ovarian cancer patients. (a)Relative expression level of SPRY4-IT1 in breast cancer and ovarian cancer samples categorized by depth of invasion (advanced stage), lymph nodes metastasis and distant metastasis. (b) Kaplan–Meier survival analysis of overall survival and disease-free survival in breast cancer and ovarian cancer patients.

**Supplementary Figure S4.** Confocal microscopy of SPRY4-IT1 localization in SW620. Cells was labeled with Cy3, DAPI. Scale bars: 10 μm. FISH was used to detect SPRY4-IT1 location in SW620 cells.

**Supplementary Figure S5.** Cells were transfected with SPRY4-IT1 cDNA or its control vector and expression levels of mRNA were determined by qRT-PCR analyses.

**Supplementary Figure S6.** STAU1 downregulation stabilized the TCEB1 mRNA in SPRY4-IT1-overexpressing HCT 116 cells. TCEB1 mRNA stability in HCT 116 cells transfected with the indicated plasmids; 48 h after transfection, the cells were treated with triptolide at a final concentration of 10 mM at the indicated times and the RNA was subsequently extracted.

**Supplementary Figure S7.** The effect of SPRY4-IT1 Alu deletion on CRC cell metastasis in vitro.

**Supplementary Figure S8.** RIP was performed to determine the specificity interaction between SPRY4-IT1 and TCEB1. (a)HCT 116 cells were transfected with full-length SPRY4-IT1, FBXL19-AS1 and LINC00346. TCEB1 levels in immunoprecipitates are presented as fold enrichment in STAU1 antibodies relative to IgG immunoprecipitates by RIP experiments. (b) HCT 116 cells were transfected with full-length SPRY4-IT1, two other Alu-containing mRNAs ANKH and RRP15 levels in immunoprecipitates are presented as fold enrichment in STAU1 antibodies relative to IgG immunoprecipitates by RIP experiments.

**Supplementary Figure S9.** Effects of SPRY4-IT1 on TCEB1 mRNA expression. SPRY4-IT1 was overexpressed in HCT 116, MCF-7 and OVCAR-3 cells (a) or knocked down in SW620, MDA-MB-231 and SK-OV-3 cancer cell lines (b). TCEB1 mRNA levels were assayed by RT-PCR and were normalized to β-actin. *p<0.05, ** p<0.01, *** p<0.001 vs. control group.

**Supplementary Figure S10.** STAU1 knock-down. qRT-PCR and Western blot analysis of STAU1 expression after STAU1 knock-down in MCF-7, HCT 116, and OVCAR-3 cell lines.

**Supplementary Figure S11.** Over-expression of STAU1. Western blot analysis of STAU1 expression after STAU1 over-expression in SW620, MDA-MB-231, and SK-OV-3 cell lines.

**Supplementary Figure S12.** TCEB1 mRNA analysis. Cells were transfected with indicated siRNAs or cDNA for 48 h and analyzed for TCEB1 mRNA expression by qRT-PCR. *p<0.05 vs. NC.

**Supplementary Figure S13.** Over-expression of TCEB1. Western blot analysis of TCEB1 expression after TCEB1 over-expression in MCF-7, HCT 116, and OVCAR-3 cell lines.

**Supplementary Figure S14.** TCEB1 knock-down. qRT-PCR and Western blot analysis of TCEB1 expression after TCEB1 knock-down in SW620, MDA-MB-231 or SK-OV-3 cell lines.

**Supplementary Figure S15.** Effects of TCEB1 siRNA on SPRY4-IT1-mediated migration and invasion in cancer cells. SW620, MDA-MB-231 or SK-OV-3 cells were co-transfected si-SPRY4-IT1 with control or TCEB1 siRNA for 48 hours, then cell migration (a) and invasion(b) were analyzed.

**Supplementary Figure S16.** The mRNA level of HIF-1α exhibited no significant change upon manipulation of SPRY4-IT1 expression in cancer cells. SPRY4-IT1 was overexpressed in HCT 116, MCF-7 and OVCAR-3 cells (a) or knocked down in SW620, MDA-MB-231 and SK-OV-3 cancer cell lines (b) HIF-1α mRNA levels were assayed by RT-PCR and were normalized to β-actin.

**Supplementary Figure S17.** Over-expression of HIF-1α. Western blot analysis of HIF-1α expression after HIF-1α over-expression in MCF-7, HCT 116, and OVCAR-3 cell lines.

**Supplementary Figure S18.** HIF-1α knock-down. qRT-PCR and Western blot analysis of HIF-1α expression after HIF-1α knock-down in MCF-7, HCT 116, and OVCAR-3 cell lines.

**Supplementary Figure S19.** Over-expression of NF-κB/p65. Western blot analysis of NF-κB/p65 expression after NF-κB/p65 over-expression in MCF-7, HCT 116, and OVCAR-3 cell lines.

**Supplementary Figure S20.** NF-κB/p65 knock-down. qRT-PCR and Western blot analysis of NF-κB/p65 expression after NF-κB/p65 knock-down in SW620, MDA-MB-231 or SK-OV-3 cell lines.

**Supplementary Figure S21.** NF-κB/p50 could not transcriptionally upregulates SPRY4-IT1. qRT-PCR analysis of SPRY4-IT1 expression in MCF-7, HCT 116 and OVCAR-3 cells following transiently transfected NF-κB/p50 cDNA (A) or in SW620, MDA-MB-231 and SK-OV-3 following transiently transfected NF-κB/p50 siRNA(B).
