# Supplementary figures and images for: NF-κB-activated SPRY4-IT1 promotes cancer cell metastasis by downregulating TCEB1 mRNA via Staufen1-mediated mRNA decay
Source: Oncogene. 2021 Jun 23;40(30):4919–29. doi: 10.1038/s41388-021-01900-8 (PMC8321898; doi:10.1038/s41388-021-01900-8)

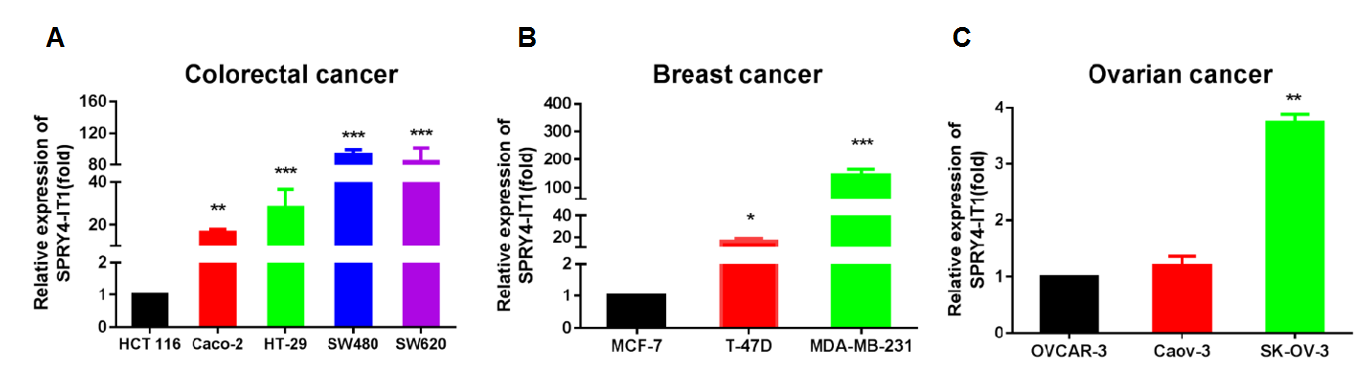

Supplement: Supplementary file 6 — Supplementary Figure S1 [file 41388_2021_1900_MOESM6_ESM.tif]

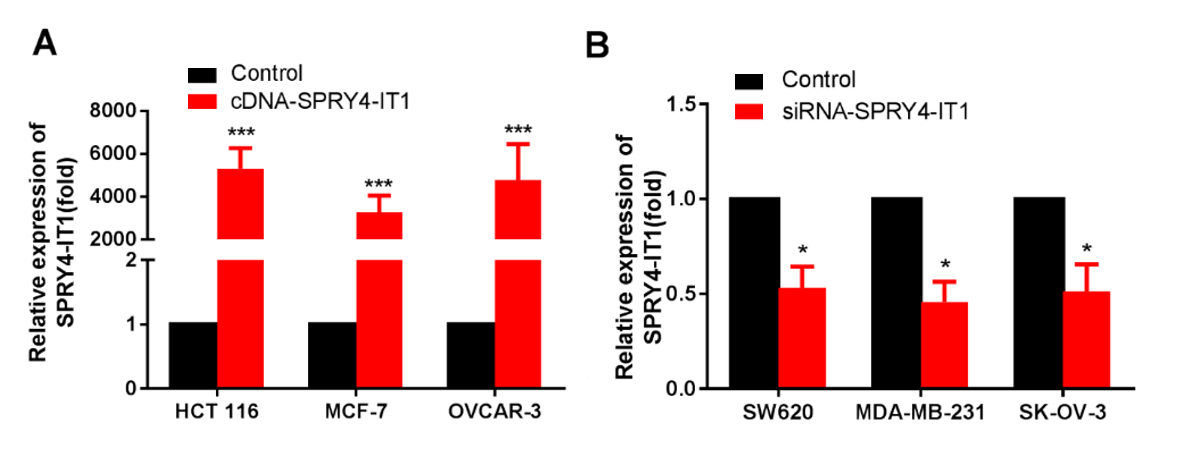

Supplement: Supplementary file 7 — Supplementary Figure S2 [file 41388_2021_1900_MOESM7_ESM.tif]

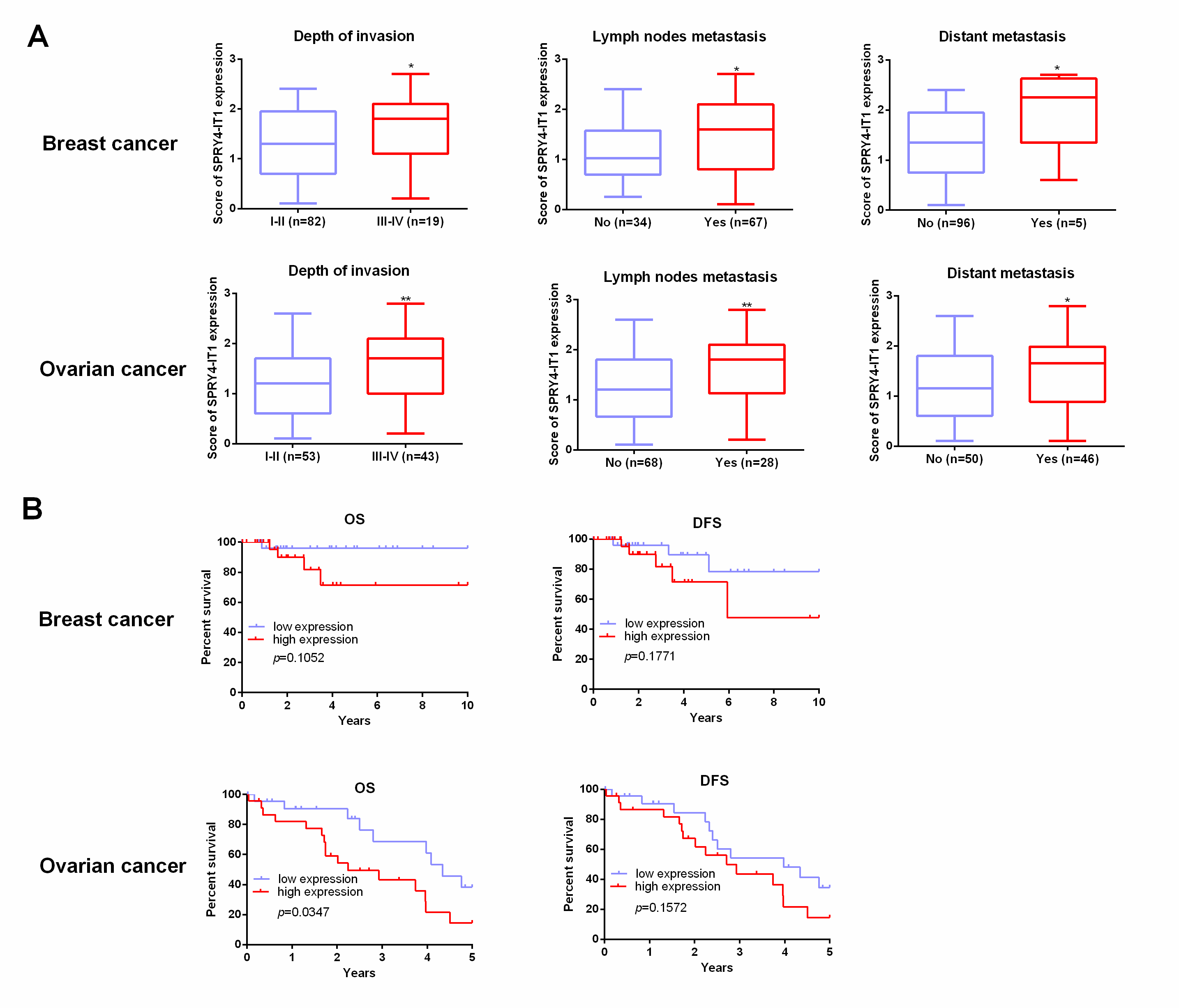

Supplement: Supplementary file 8 — Supplementary Figure S3 [file 41388_2021_1900_MOESM8_ESM.tif]

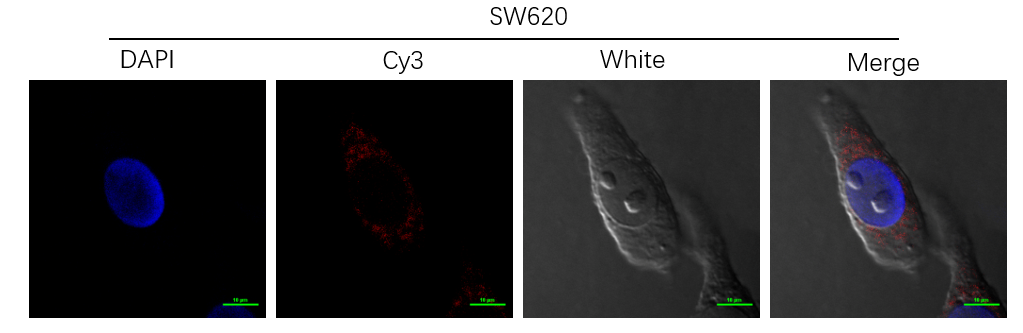

Supplement: Supplementary file 9 — Supplementary Figure S4 [file 41388_2021_1900_MOESM9_ESM.tif]

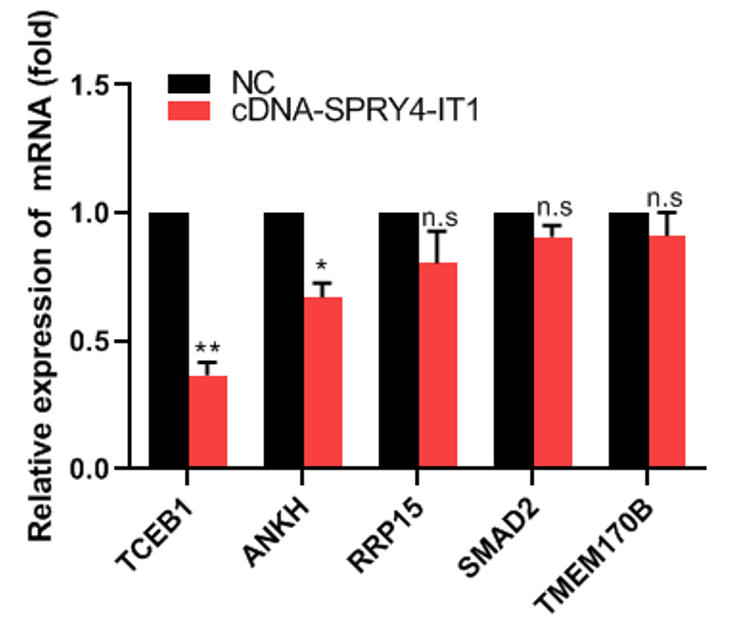

Supplement: Supplementary file 10 — Supplementary Figure S5 [file 41388_2021_1900_MOESM10_ESM.tif]

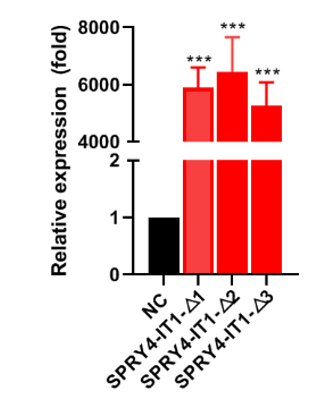

Supplement: Supplementary file 11 — Supplementary Figure S6 [file 41388_2021_1900_MOESM11_ESM.tif]

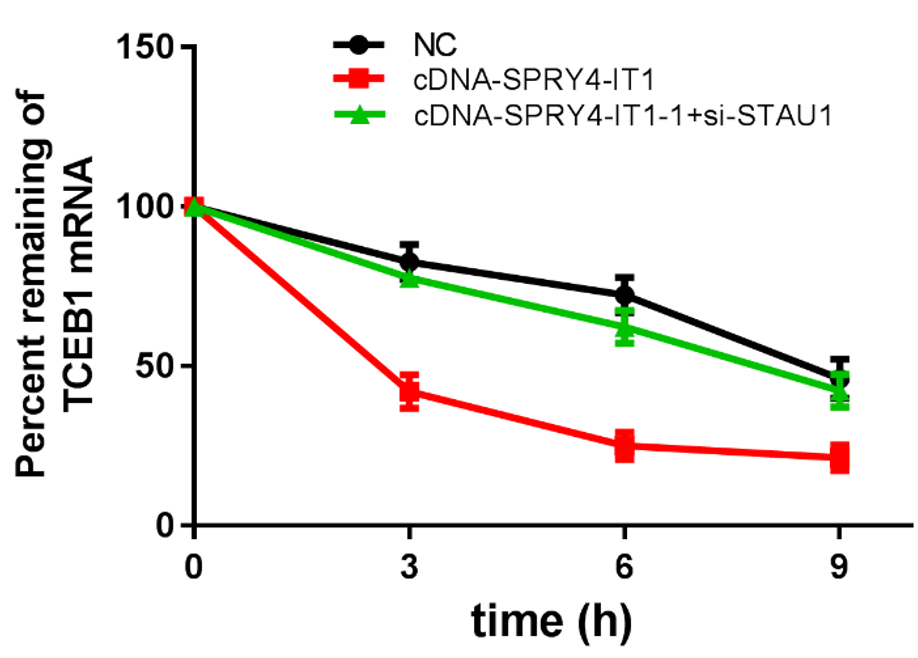

Supplement: Supplementary file 12 — Supplementary Figure S7 [file 41388_2021_1900_MOESM12_ESM.tif]

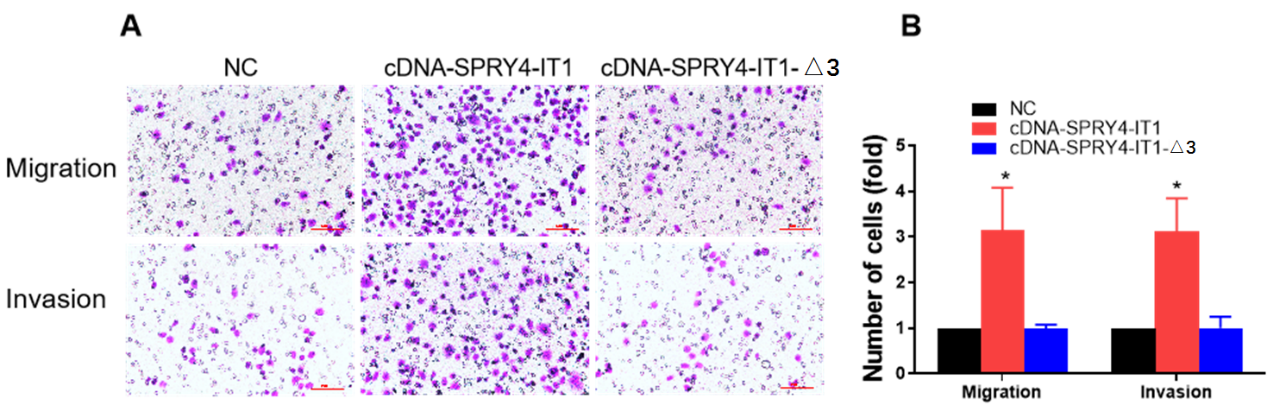

Supplement: Supplementary file 13 — Supplementary Figure S8 [file 41388_2021_1900_MOESM13_ESM.tif]

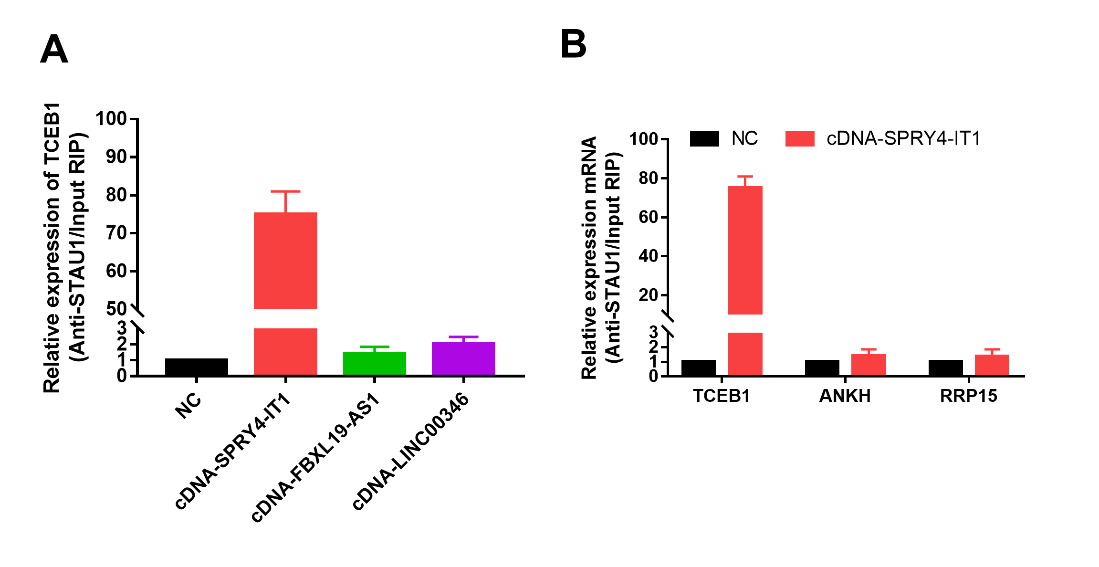

Supplement: Supplementary file 14 — Supplementary Figure S9 [file 41388_2021_1900_MOESM14_ESM.tif]

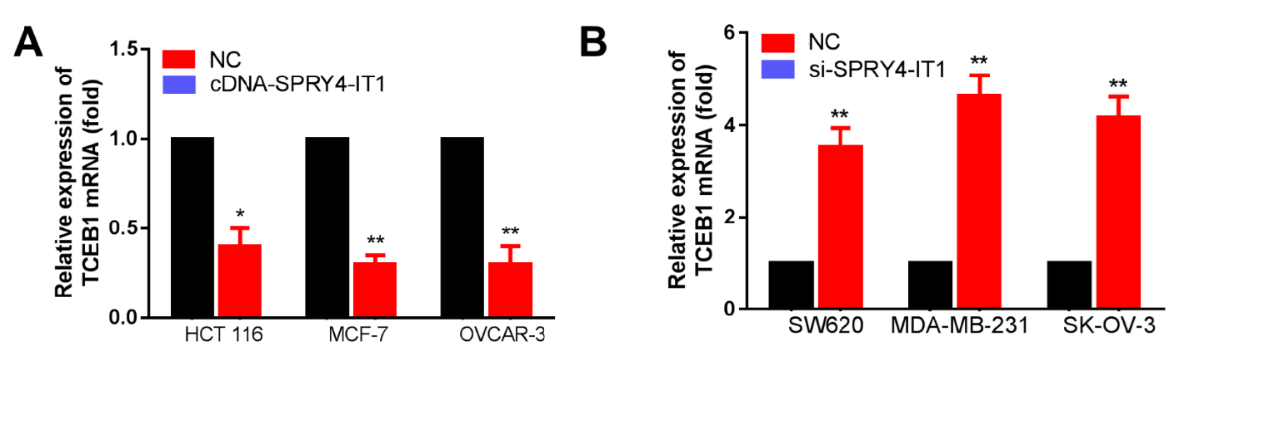

Supplement: Supplementary file 15 — Supplementary Figure S10 [file 41388_2021_1900_MOESM15_ESM.tif]

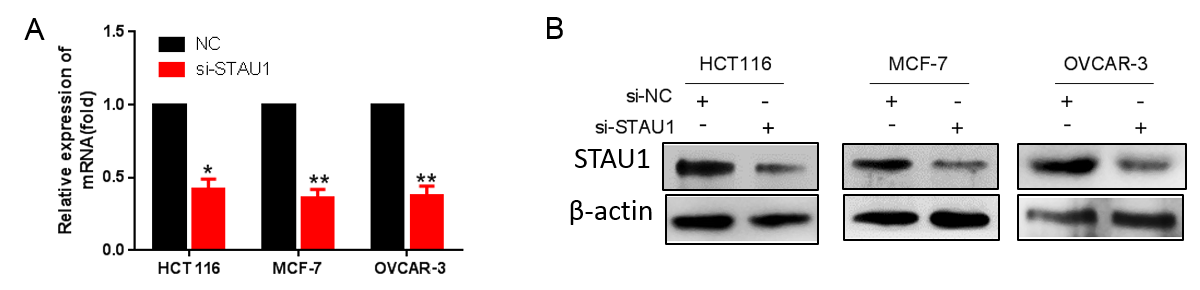

Supplement: Supplementary file 16 — Supplementary Figure S11 [file 41388_2021_1900_MOESM16_ESM.tif]

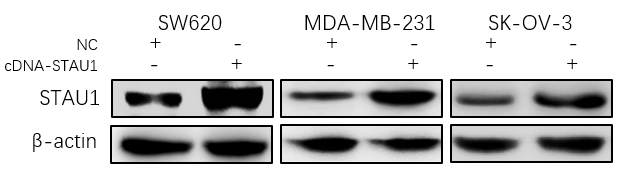

Supplement: Supplementary file 17 — Supplementary Figure S12 [file 41388_2021_1900_MOESM17_ESM.tif]

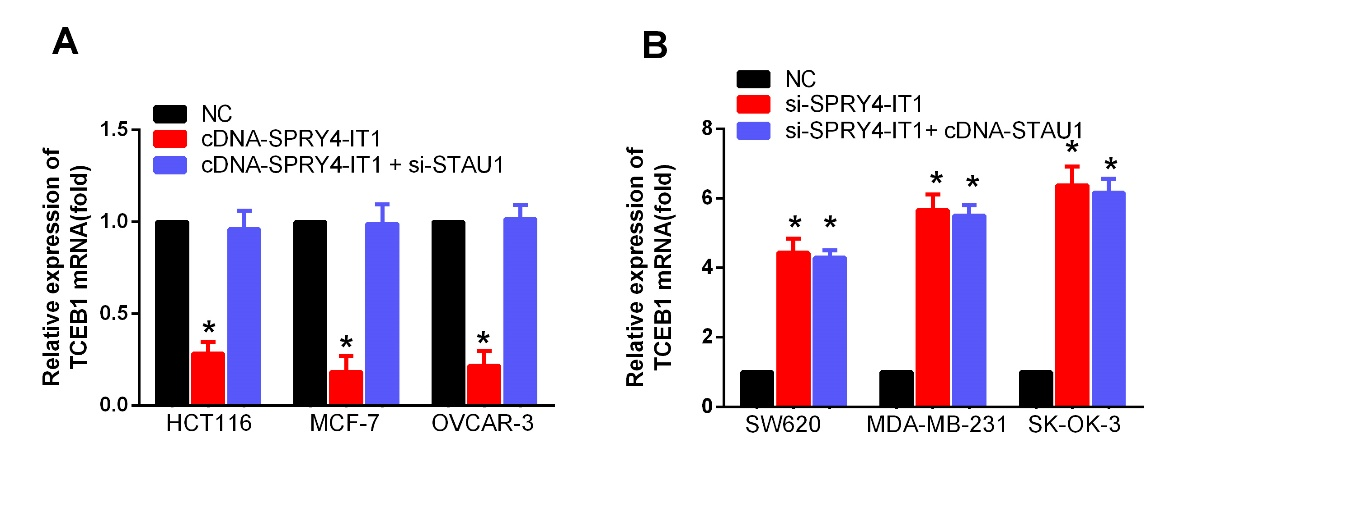

Supplement: Supplementary file 18 — Supplementary Figure S13 [file 41388_2021_1900_MOESM18_ESM.tif]

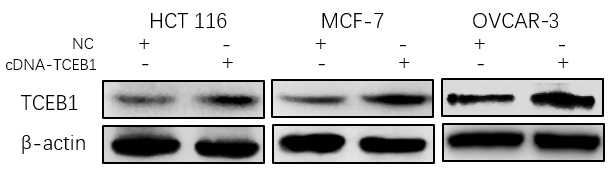

Supplement: Supplementary file 19 — Supplementary Figure S14 [file 41388_2021_1900_MOESM19_ESM.tif]

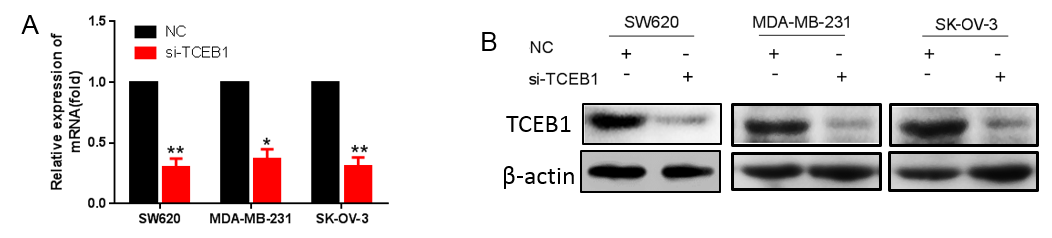

Supplement: Supplementary file 20 — Supplementary Figure S15 [file 41388_2021_1900_MOESM20_ESM.tif]

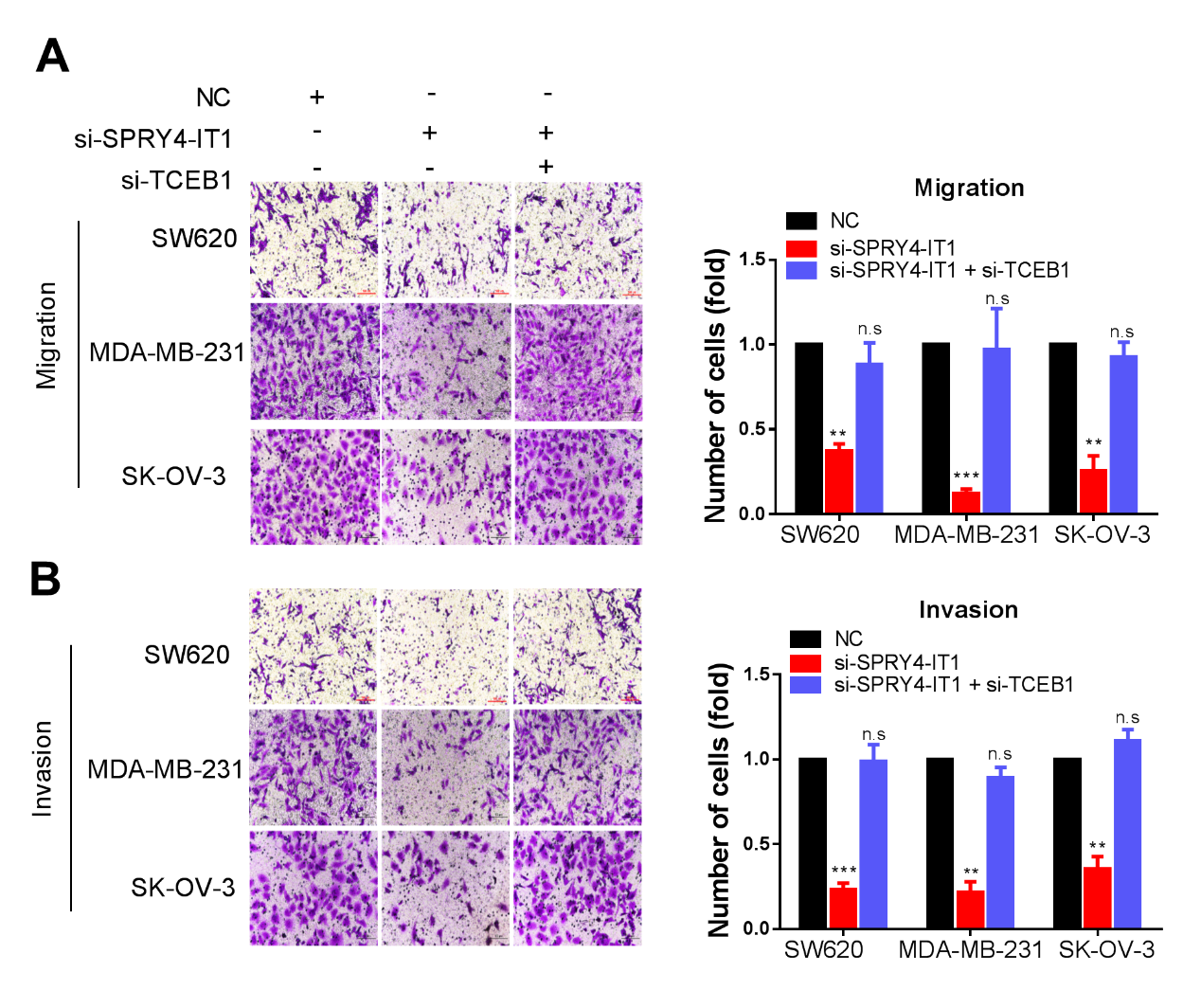

Supplement: Supplementary file 21 — Supplementary Figure S16 [file 41388_2021_1900_MOESM21_ESM.tif]

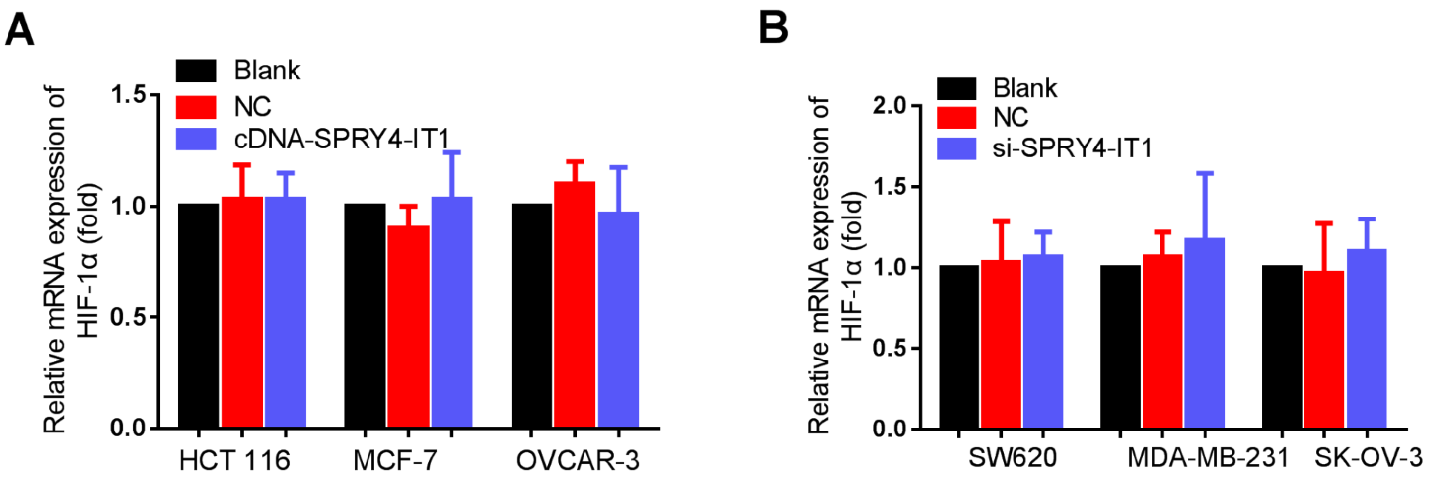

Supplement: Supplementary file 22 — Supplementary Figure S17 [file 41388_2021_1900_MOESM22_ESM.tif]

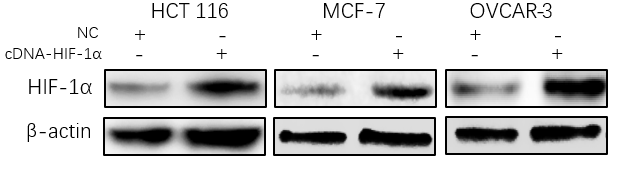

Supplement: Supplementary file 23 — Supplementary Figure S18 [file 41388_2021_1900_MOESM23_ESM.tif]

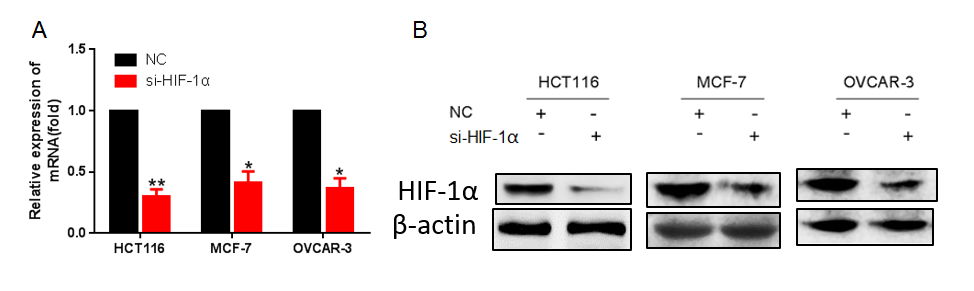

Supplement: Supplementary file 24 — Supplementary Figure S19 [file 41388_2021_1900_MOESM24_ESM.tif]

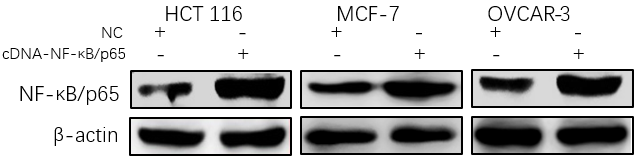

Supplement: Supplementary file 25 — Supplementary Figure S20 [file 41388_2021_1900_MOESM25_ESM.tif]

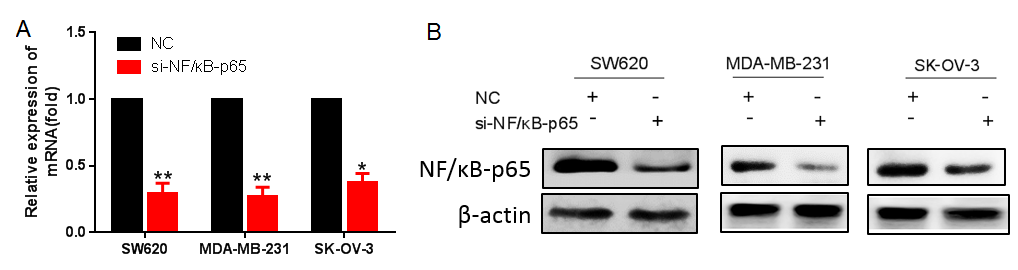

Supplement: Supplementary file 26 — Supplementary Figure S21 [file 41388_2021_1900_MOESM26_ESM.tif]

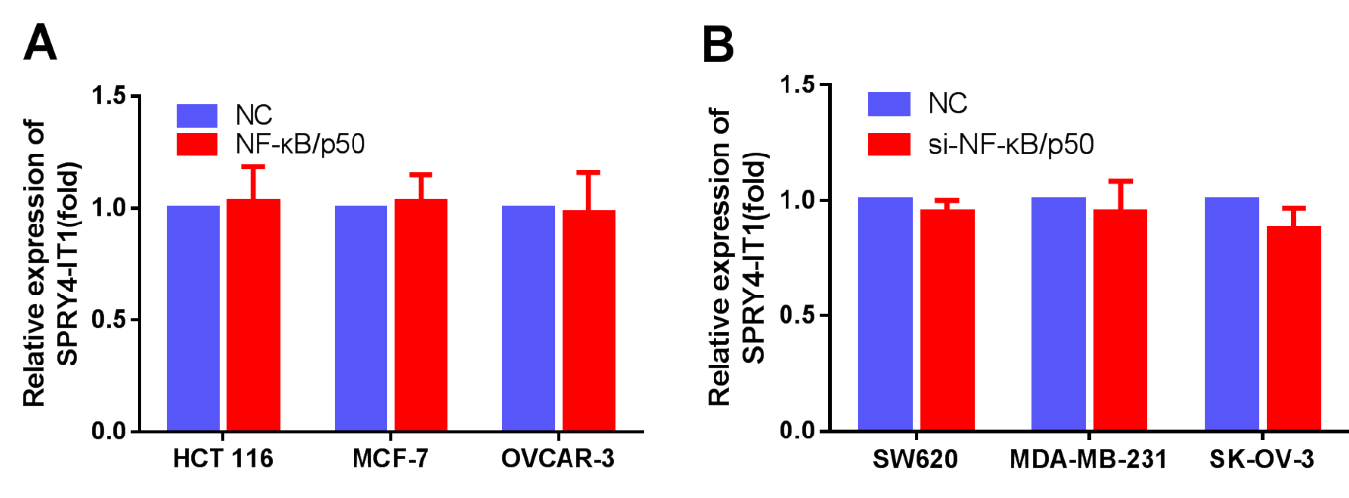

Supplement: Supplementary file 27 — Supplementary Figure S22 [file 41388_2021_1900_MOESM27_ESM.tif]
